# Supplementary material for: Carbonizing technology enables Sanguisorbae Radix to inhibit yeast-to-hypha differentiation and biofilm formation in Candida albicans
Source: PLoS One. 2025 Oct 17;20(10):e0334659. doi: 10.1371/journal.pone.0334659 (PMC12533860; doi:10.1371/journal.pone.0334659)

**S1 Fig. Comparison of the inhibitory effects of SR extract and CSR extract on *C. albicans*.** (A) Validation of the antifungal effects of SR extract and CSR extract on *C. albicans* at concentrations of 20, 100 and 500 μg/mL. CSR exhibited superior inhibitory activity compared to SR at concentrations of 100 and 500 μg/mL. Compared with the control group, *** *P*<0.001, ** *P*<0.01, * *P*<0.05. (B) Verification of growth status of *C. albicans* of CSR extract at concentrations of 100 μg/mL in SDA culture medium. CSR showed significantly stronger inhibitory effects against *C. albicans* at 100 μg/mL than SR. (C) The effects of SR extract and CSR extract on the microscopic morphology of *C. albicans* in 100 μg/mL. The cell membrane of *C. albicans* in the control group was intact, smooth, and well-defined. In the SR-treated group, mucus-like substances were observed between *C. albicans* cells, but no significant structural changes in the fungal cells were noted. In contrast, the CSR-treated group exhibited substantial cell wrinkling and deformation, with approximately half of the fungal cells rupturing and showing cytoplasmic leakage. All groups n=3. All control groups contained DMSO without extract.


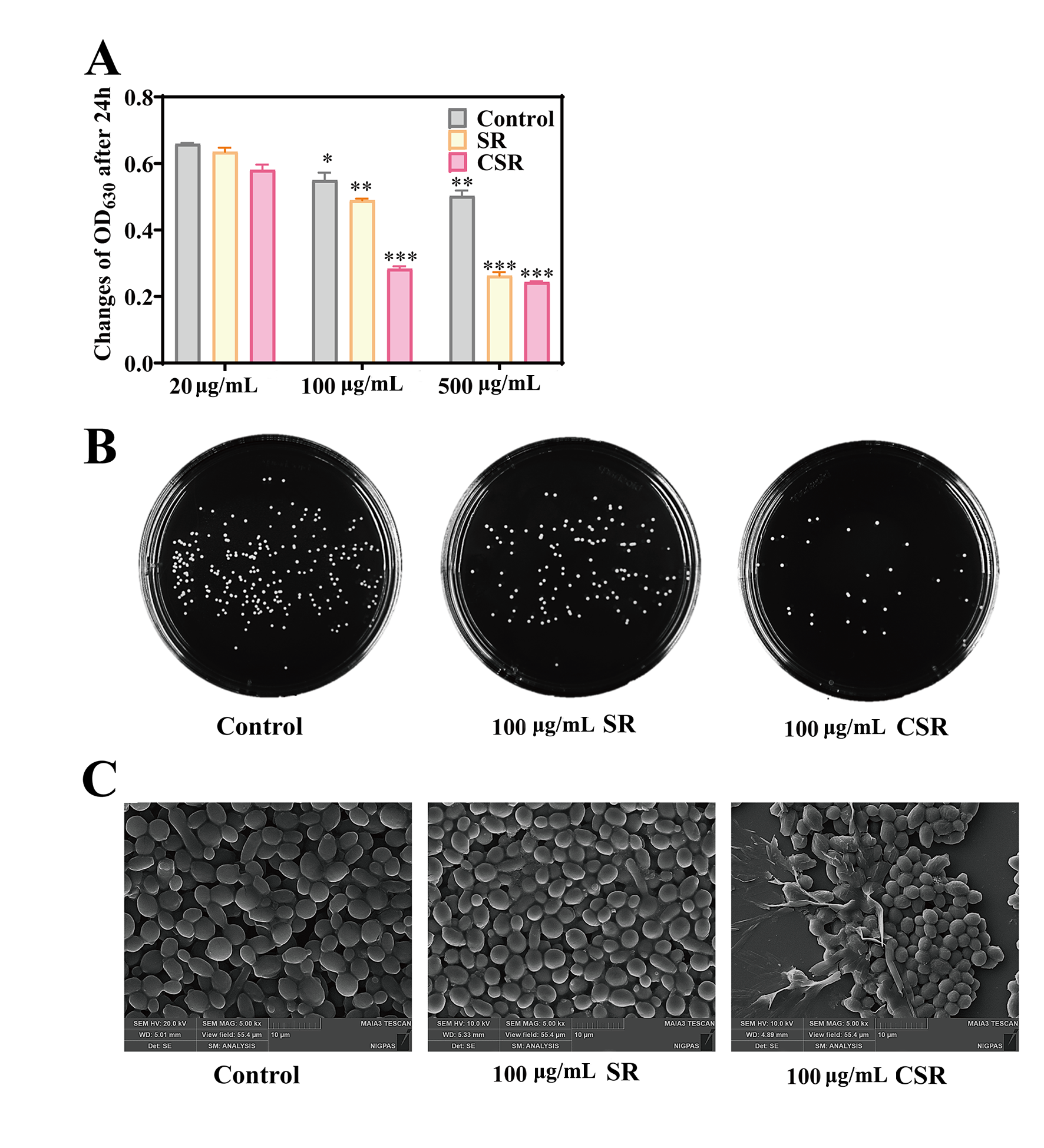

Supplement: S1 Fig — (A) Validation of the antifungal effects of SR extract and CSR extract on C. albicans at concentrations of 20, 100 and 500 μg/mL. CSR exhibited superior inhibitory activity compared to SR at concentrations of 100 and 500 μg/mL. Compared with the control group, *** P < 0.001, ** P < 0.01, * P < 0.05. (B) Verification of growth status of C. albicans of CSR extract at concentrations of 100 μg/mL in SDA culture medium. CSR showed significantly stronger inhibitory effects against C. albicans at 100 μg/mL than SR. (C) The effects of SR extract and CSR extract on the microscopic morphology of C. albicans in 100 μg/mL. The cell membrane of C. albicans in the control group was intact, smooth, and well-defined. In the SR-treated group, mucus-like substances were observed between C. albicans cells, but no significant structural changes in the fungal cells were noted. In contrast, the CSR-treated group exhibited substantial cell wrinkling and deformation, with approximately half of the fungal cells rupturing and showing cytoplasmic leakage. All groups n = 3. All control groups contained DMSO without extract. (DOCX) [file pone.0334659.s001.docx]
