# Supplementary material for: Carbonizing technology enables Sanguisorbae Radix to inhibit yeast-to-hypha differentiation and biofilm formation in Candida albicans
Source: PLoS One. 2025 Oct 17;20(10):e0334659. doi: 10.1371/journal.pone.0334659 (PMC12533860; doi:10.1371/journal.pone.0334659)

**S2 Fig. Evaluation of the antifungal mechanism of CSR extract against *C. albicans*.** (A) The effect of CSR extract (100 μg/mL) on the cell membrane of *C. albicans*. Ergosterol, the primary component of the *C. albicans* cell membrane, was found to decrease in content after incubation with CSR. Compared with the control group, *** *P*<0.001, ** *P*<0.01. (B) Cell permeability of *C. albicans* after incubation with CSR extract (100 μg/mL). A marked increase in protein content in the supernatant of *C. albicans* incubated with CSR following vortexing. Compared with the control group, *** *P*<0.001. (C) The effect of CSR extract (100 μg/mL) on the cell wall of *C. albicans*. The concentrations of fluconazole and caspofungin used as positive and negative controls were 10 μg/mL and 1 μg/mL. The main component of the *C. albicans* cell wall is β-1,3-glucan, which can be protected by sorbitol. When sorbitol was added to the culture medium, CSR continued to exhibit inhibitory effects. This indicated that CSR does not affect the synthesis of the *C. albicans* cell wall. Compared with the control group, ** *P*<0.01. (D) Comparison of the inhibitory effect of CSR extract (100 μg/mL) on *C. albicans* in SDB medium with different pH values. The inhibitory activity of CSR against *C. albicans* was dependent on pH levels. All groups n=3. All control groups contained DMSO without extract.


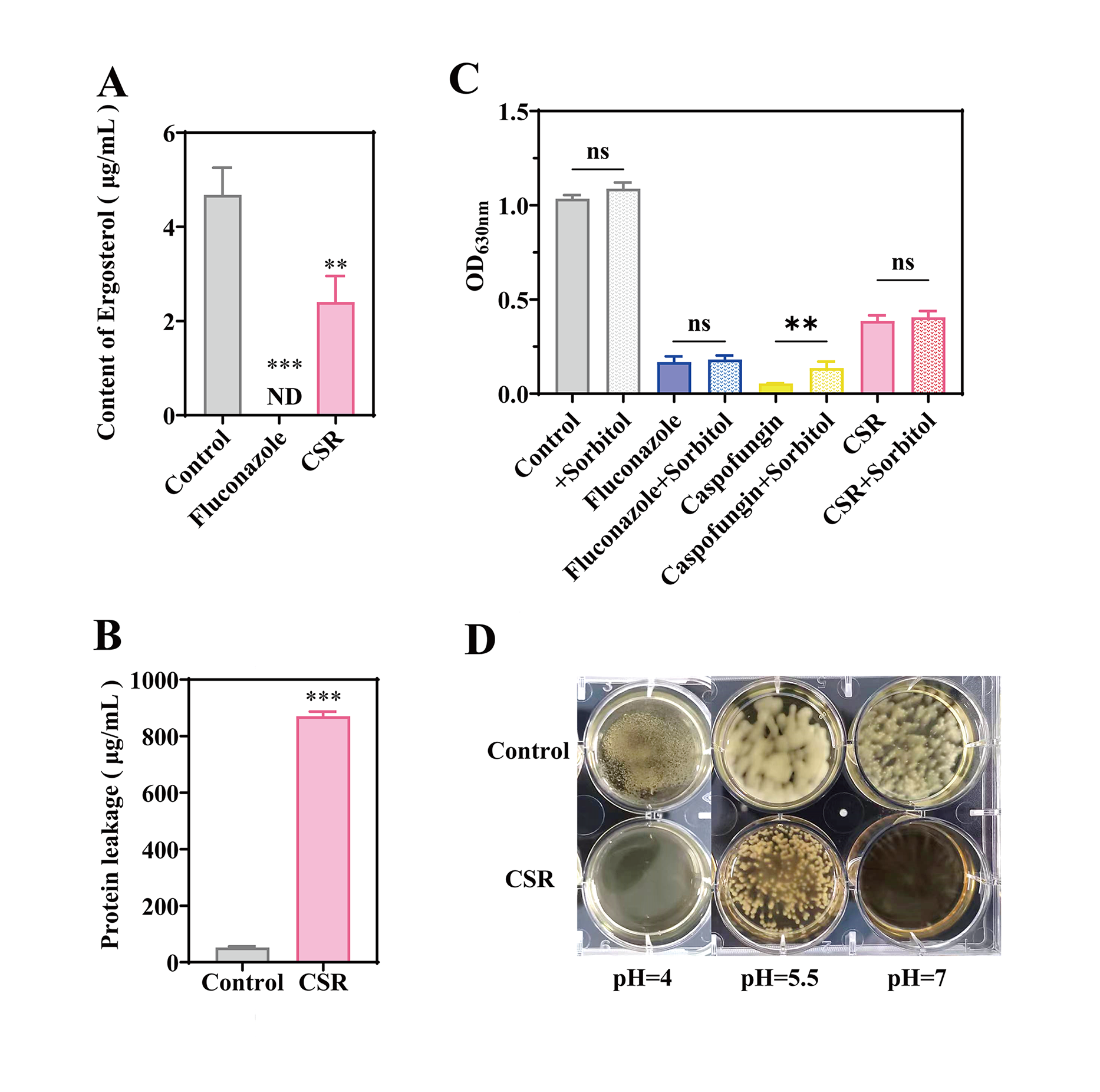

Supplement: S2 Fig — (A) The effect of CSR extract (100 μg/mL) on the cell membrane of C. albicans. Ergosterol, the primary component of the C. albicans cell membrane, was found to decrease in content after incubation with CSR. Compared with the control group, *** P < 0.001, ** P < 0.01. (B) Cell permeability of C. albicans after incubation with CSR extract (100 μg/mL). A marked increase in protein content in the supernatant of C. albicans incubated with CSR following vortexing. Compared with the control group, *** P < 0.001. (C) The effect of CSR extract (100 μg/mL) on the cell wall of C. albicans. The concentrations of fluconazole and caspofungin used as positive and negative controls were 10 μg/mL and 1 μg/mL. The main component of the C. albicans cell wall is β-1,3-glucan, which can be protected by sorbitol. When sorbitol was added to the culture medium, CSR continued to exhibit inhibitory effects. This indicated that CSR does not affect the synthesis of the C. albicans cell wall. Compared with the control group, ** P < 0.01. (D) Comparison of the inhibitory effect of CSR extract (100 μg/mL) on C. albicans in SDB medium with different pH values. The inhibitory activity of CSR against C. albicans was dependent on pH levels. All groups n = 3. All control groups contained DMSO without extract. (DOCX) [file pone.0334659.s002.docx]
