# Supplementary material for: Carbonizing technology enables Sanguisorbae Radix to inhibit yeast-to-hypha differentiation and biofilm formation in Candida albicans
Source: PLoS One. 2025 Oct 17;20(10):e0334659. doi: 10.1371/journal.pone.0334659 (PMC12533860; doi:10.1371/journal.pone.0334659)

**S3 Fig. Comparison of physical parameters and chemical composition between SR and CSR.** (A) Pieces, sections, and powder diagrams of SR and CSR. The surface, cross-section, and powder of SR are uniformly yellow, whereas those of CSR are uniformly black, with some pieces appearing ashed. (B) The content of total tannins, condensed tannins, and hydrolysable tannins in SR and CSR. Condensed tannins were undetectable after charcoal frying. (C) The difference between ellagitannin and gallotannin in hydrolyzed tannins. Hydrolyzed tannins in both SR and CSR predominantly consisted of ellagitannins and gallotannins. Charcoal frying induced notable compositional changes, with a significant increase in ellagitannin content observed in CSR. Compared with the SR group, *** *P*<0.001. All groups n=3.


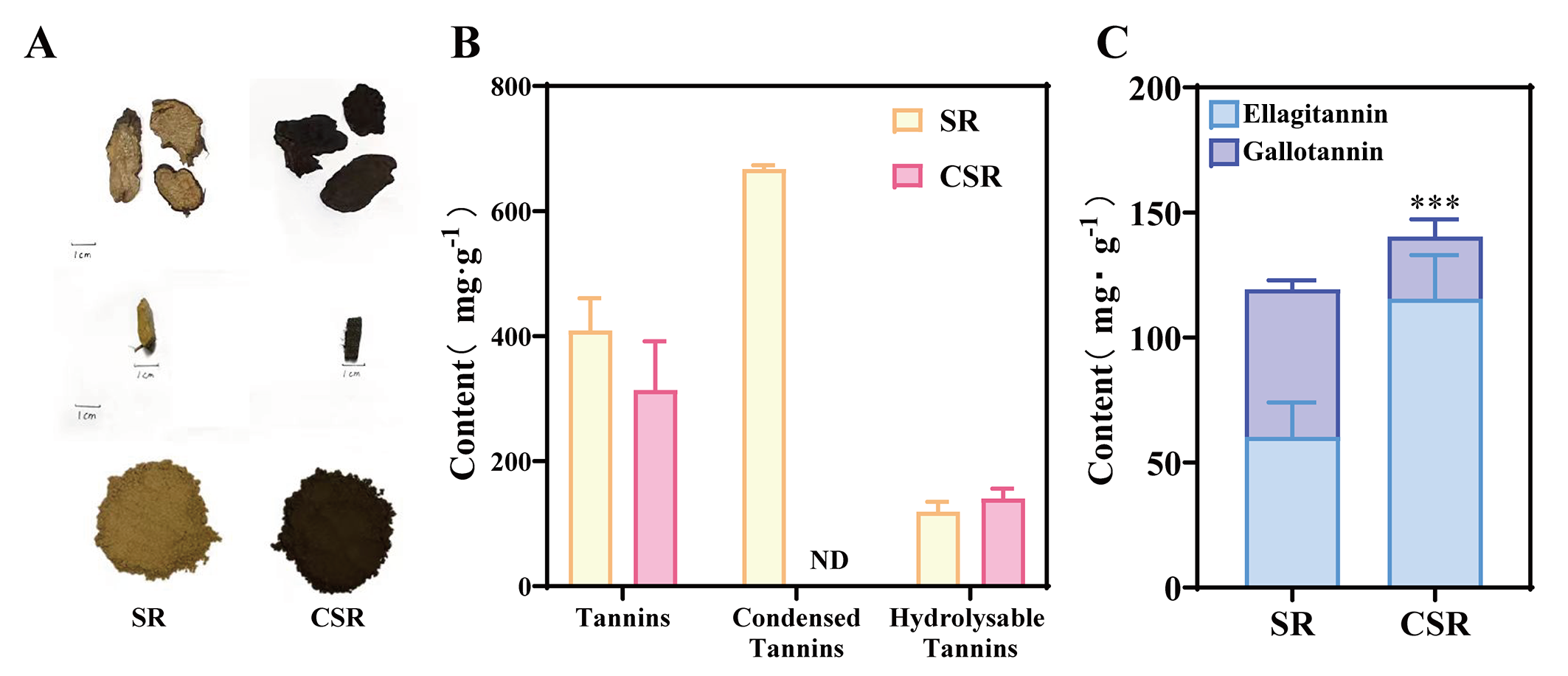

Supplement: S3 Fig — (A) Pieces, sections, and powder diagrams of SR and CSR. The surface, cross-section, and powder of SR are uniformly yellow, whereas those of CSR are uniformly black, with some pieces appearing ashed. (B) The content of total tannins, condensed tannins, and hydrolysable tannins in SR and CSR. Condensed tannins were undetectable after charcoal frying. (C) The difference between ellagitannin and gallotannin in hydrolyzed tannins. Hydrolyzed tannins in both SR and CSR predominantly consisted of ellagitannins and gallotannins. Charcoal frying induced notable compositional changes, with a significant increase in ellagitannin content observed in CSR. Compared with the SR group, *** P < 0.001. All groups n = 3. (DOCX) [file pone.0334659.s003.docx]
