# Supplementary material for: Carbonizing technology enables Sanguisorbae Radix to inhibit yeast-to-hypha differentiation and biofilm formation in Candida albicans
Source: PLoS One. 2025 Oct 17;20(10):e0334659. doi: 10.1371/journal.pone.0334659 (PMC12533860; doi:10.1371/journal.pone.0334659)

**S4 Fig. Mass spectrometry information of compounds in CSR extract.** (A) Ellagic acid. (B) Gallic acid. (C) Pyrogallic acid. (D) Methyl Gallate. (E) Protocatechuic Acid. (F) Catechin.


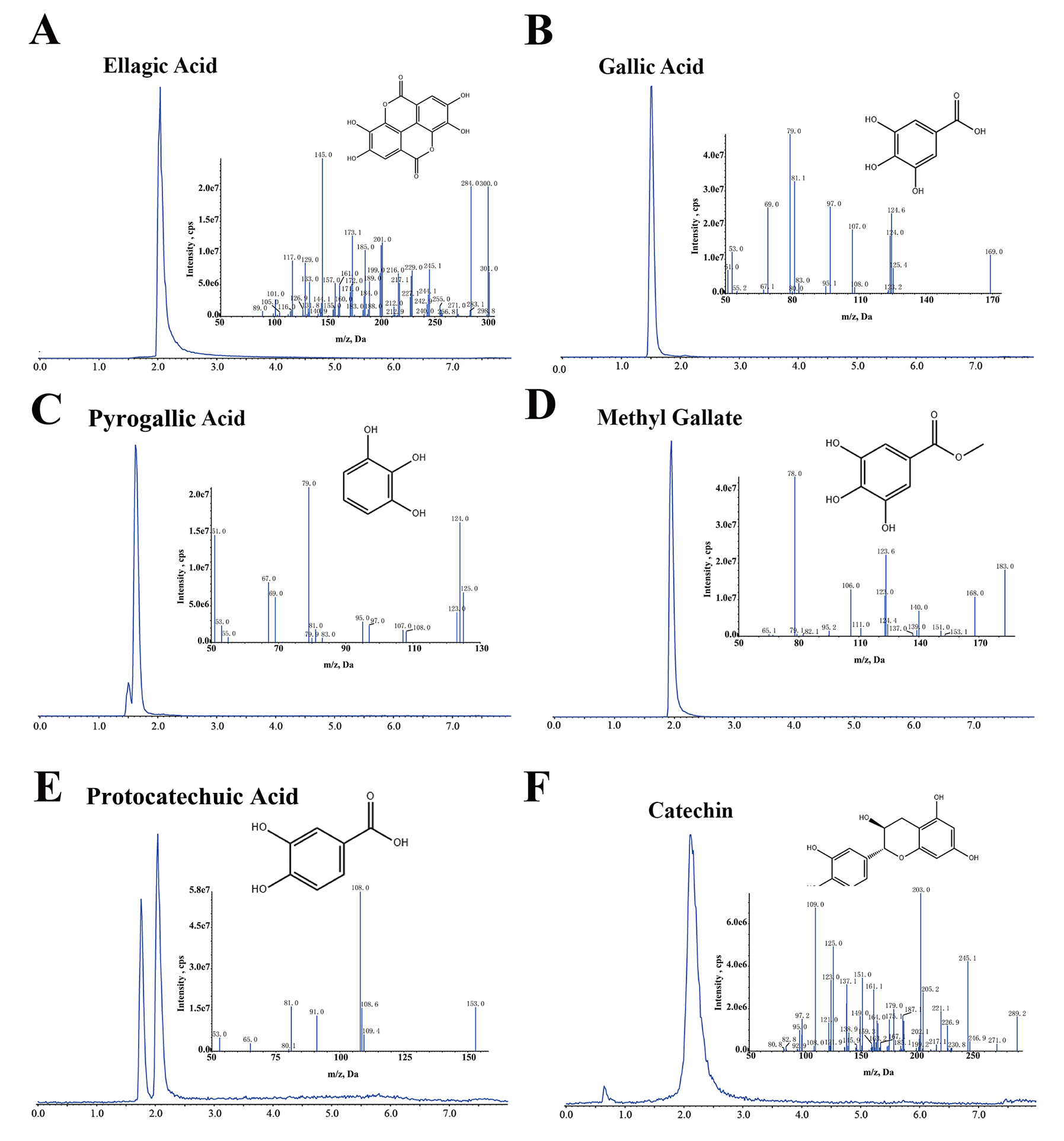

Supplement: S4 Fig — (A) Ellagic acid. (B) Gallic acid. (C) Pyrogallic acid. (D) Methyl Gallate. (E) Protocatechuic Acid. (F) Catechin. (DOCX) [file pone.0334659.s004.docx]
