# Supplementary material for: Carbonizing technology enables Sanguisorbae Radix to inhibit yeast-to-hypha differentiation and biofilm formation in Candida albicans
Source: PLoS One. 2025 Oct 17;20(10):e0334659. doi: 10.1371/journal.pone.0334659 (PMC12533860; doi:10.1371/journal.pone.0334659)

**S5 Fig. Study on the efficacy of fluconazole in treating VVC.** HE staining (A) and PAS staining (B) of mouse vaginal tissue. After fluconazole treatment, there was no significant keratinization or congestion in the submucosal tissue and marked improvement in inflammation. However, some hyphae remained attached to the vaginal wall.


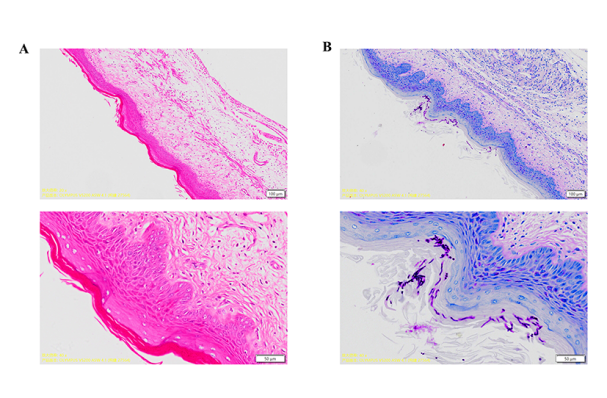

Supplement: S5 Fig — HE staining (A) and PAS staining (B) of mouse vaginal tissue. After fluconazole treatment, there was no significant keratinization or congestion in the submucosal tissue and marked improvement in inflammation. However, some hyphae remained attached to the vaginal wall. (DOCX) [file pone.0334659.s005.docx]
