# Supplementary material for: Carbonizing technology enables Sanguisorbae Radix to inhibit yeast-to-hypha differentiation and biofilm formation in Candida albicans
Source: PLoS One. 2025 Oct 17;20(10):e0334659. doi: 10.1371/journal.pone.0334659 (PMC12533860; doi:10.1371/journal.pone.0334659)
Supplement: S1 Table — (DOCX) [file pone.0334659.s006.docx]

**S1 Table. The ancient Chinese documents records the treatment of fungal vaginitis with SR.**

| Ancient documents | Dynasty | The content recorded |
| --- | --- | --- |
| Shennong's classic of materia medica | Eastern Han Dynasty | Treating women with breast pain, seven injuries, and twelve underlying diseases, providing pain relief… |
| Tang materia medica | Tang Dynasty | The twelve diseases under the main belt |
| Compendium of Materia Medica | Ming dynasty | And the main red and white belt |
| Synopsis of Treating Women's Diseases | Ming dynasty | Five colors for treating leaks, eleven or twelve belts |
| Standards of Diagnosis and Treatment | Ming dynasty | Treat those with red and white discharge and bone erection |
| Continued Commentary on Materia Medica | Ming dynasty | If the SR is not treated, it will lead to its downfall…Only able to relieve pain for seven injuries and underlying illnesses |
| Herbal Justice | Qing dynasty | Treating breast pain in women |
| Materia medica Fengyuan | Qing dynasty | Main seven injuries and five leaks |
