# Supplementary material for: Carbonizing technology enables Sanguisorbae Radix to inhibit yeast-to-hypha differentiation and biofilm formation in Candida albicans
Source: PLoS One. 2025 Oct 17;20(10):e0334659. doi: 10.1371/journal.pone.0334659 (PMC12533860; doi:10.1371/journal.pone.0334659)
Supplement: S2 Table — (DOCX) [file pone.0334659.s007.docx]

**S2 Table.** **MIC of SR extract, CSR extract and related components against *C. albicans.***

| Drug | MIC ( μg/mL ) | | |
| --- | --- | --- | --- |
| SR extract | 1024 | 1024 | 1024 |
| CSR extract | 1024 | 1024 | 1024 |
| Ellagic Acid | 256 | 256 | 256 |
| Gallic Acid | 512 | 512 | 512 |
| Catechin | 256 | 256 | 256 |
| Pyrogallic Acid | 512 | 512 | 512 |
| Fluconazole | 15.6 | 15.6 | 15.6 |
| Clotrimazole | 7.8 | 7.8 | 7.8 |
