# Supplementary material for: Carbonizing technology enables Sanguisorbae Radix to inhibit yeast-to-hypha differentiation and biofilm formation in Candida albicans
Source: PLoS One. 2025 Oct 17;20(10):e0334659. doi: 10.1371/journal.pone.0334659 (PMC12533860; doi:10.1371/journal.pone.0334659)
Supplement: S3 Table — (DOCX) [file pone.0334659.s008.docx]

**S3 Table. Comparison of Physical Parameters Related to SR and CSR.**

| Specimen | Yield Rate by Charring（%） | Extraction yield（%） | Texture | Average Value of Powder Color Response | | | |
| --- | --- | --- | --- | --- | --- | --- | --- |
|  |  |  |  | L* | a* | b* | ΔE*ab |
| SR | / | 32.13±3.63 | Hard, not easy to break | 58.21 | 8.05 | 21.28 | / |
| CSR | 65.86±6.73 | 7.98±0.78 | Loose and brittle, partially ashed | 29.89 | 1.77 | -1.74 | 685.77 |
