# Supplementary material for: Carbonizing technology enables Sanguisorbae Radix to inhibit yeast-to-hypha differentiation and biofilm formation in Candida albicans
Source: PLoS One. 2025 Oct 17;20(10):e0334659. doi: 10.1371/journal.pone.0334659 (PMC12533860; doi:10.1371/journal.pone.0334659)
Supplement: Attachment — Submitted filename: Response to reviewers.docx [file pone.0334659.s010.docx]

|  |  |  | The Second Hospital of Nanjing, Affiliated to Nanjing University of Chinese Medicine  1-1 Zhongfu Road  Nanjing 210003 China  Telephone +86 (0)25 8519 8648  Email: njdie001@njucm.edu.cn |
| --- | --- | --- | --- |
|  |  | |  |
|  | **Hongyu Zhao** | |  |

##### 17 June 2025

##### Manuscript Number: PONE-D-25-10562

**Carbonizing Technology Enables *Sanguisorbae Radix* to Inhibit Yeast-to-Hypha Differentiation and Biofilm Formation in *Candida albicans***

Dear Editor,

Thank you for the opportunity to respond to Reviewers’ comments on our manuscript. We have revised the manuscript and the figures according to reviewer’s comments. The changes in the text are highlighted in red. I hope that the changes are adequate for acceptance of the manuscript for publication in PLOS ONE.

Yours sincerely,

Hongyu Zhao, on behalf of all authors

Comments to the Author 

Reviewer #1: The research has been conducted well with sound methodology and interesting results.

However, the 'Conclusion' is unsatisfactory and I suggest to re-write it - conclusion should be based on the findings of your research. Avoid general comments and be specific on what you conclude from your results. Restate your main findings, may add future directions to improve the knowledge. Further, do not include references in the conclusion.

**Response:** We thank Reviewer 1 for the comments. We have revised the Conclusion section to better reflect the main findings of our research. The updated ‘conclusion’ can be found on lines 575-582, with all changes highlighted in red.

Reviewer #2: The manuscript entitled “Carbonizing Technology Enables Sanguisorbae Radix to Inhibit Yeast-to-Hypha Differentiation and Biofilm Formation in Candida albicans” provides a detailed account of the ability of charred Sanguisorbae Radix (CSR) to inhibit the yeast-to-hypha transition and biofilm formation in C. albicans. Although the manuscript is impressive, several minor suggestions and corrections are necessary to improve clarity and scientific rigor. Based on the following points, I recommend major revision:

1. In the Methodology section titled “Research on the inhibitory effect of biofilms on C. albicans*,” the authors should clearly mention the concentration of the compound added during the assay.

**Response:** We thank Reviewer 2 for the comments. We have added the concentrations of the compounds, including SR extract (20 μg/mL or 100 μg/mL) and CSR extract (20 μg/mL or 100 μg/mL), in the section titled ‘Inhibitory effect of pre-established biofilms on *C. albicans*’ on lines 205-206. The revisions have been highlighted in red.

2. In the same section, it appears that the assay was performed on pre-formed biofilms of C. albicans. If this is the case, the authors should revise the title of the methodology to reflect that the study investigates the effect on pre-established biofilms, not the initial biofilm formation.

**Response:** Thank you for the comments. We have revised the title on line 201 to ‘Inhibitory of pre-established biofilms on *C. albicans*’.

3. The authors should clarify the impact of SR and CSR on the viability of C. albicans. This will help differentiate between anti-biofilm effects and possible antifungal (fungicidal or fungistatic) activity.

**Response:** Thank you for the comments. We have clarified the effects of SR and CSR on the viability of *C. albicans* in S1_File. The minimum inhibitory concentrations (MICs) of both SR and CSR extracts against *C. albicans* were measured at 1024 μg/mL (S2 Table). As shown in Fig 2A, doses of SR and CSR at 20 or 100 μg/mL significantly reduced biofilm formation. Therefore, SR and CSR affect both the biofilm formation of *C. albicans* and antifungal activity.

4. Although the authors have explained the results of the transcriptomic analysis well, a brief summary of the major transcriptomic findings should be incorporated into the Discussion section to strengthen the overall interpretation of the data.

**Response:** Thank you for the comments. We have added a brief summary of the main transcriptomic findings to the ‘Discussion’ section on lines 487-489. The changes have been highlighted in red.

5. In the methodology section titled “The effect of CSR on the expression of pathogenic genes of C. albicans*,” the authors should specify the concentration of CSR used, the time point of treatment, and the duration of incubation. This information is essential, especially since biofilm assays were conducted at various time points.

**Response:** Thank you for the comments. We have included the concentration of CSR extract (100 μg/mL), the treatment time point (0 hours), and the incubation duration (24 hours) in the methodology section titled ‘The effect of CSR on the expression of pathogenic genes of C. albicans’ on lines 252-253.

6. The authors are advised to italicize the terms “in vitro” and “in vivo” throughout the manuscript, in accordance with standard scientific formatting.

**Response:** Thank you for the comments. We have revised the manuscript to italicize the terms in vitro and in vivo, in accordance with standard scientific formatting conventions.

7. The authors should discuss the possible mechanism underlying the enhanced content of ellagic acid (EA), gallic acid (GR), and pyrogallic acid (PYG) in SR following carbonization to produce CSR.

**Response:** We appreciate this comment. The mechanism underlying the increased EA, GA, and PYG content in carbonized SR (CSR) was indeed elucidated in our prior publication (PMID: 35309515). As detailed in Lines 520-524 of the Discussion, stir-frying carbonization cleaves hydrolyzable tannins' glycosidic/ester bonds to liberate GA, which subsequently converts to PYG, while simultaneously hydrolyzing ellagitannins into EA derivatives. This dual transformation pathway explains the marked PYG elevation in CSR.

8. In the sentence“To further validate EA’s contribution to CSR’s antifungal efficacy, an equivalent amount of EA was added to the CSR extract,”it should be clarified whether similar experiments were conducted with GR and PYG. If so, the authors should describe the impact of adding these compounds to CSR and explain how the results led to the conclusion that EA exhibits the strongest antifungal activity.

**Response:** Thank you for the comments. While we focused on EA due to its substantially higher concentration in CSR (34.3 mg/g) compared to GA (9.82 mg/g) and PYG (6.46 mg/g) (Figure 4A), our preliminary testing also demonstrated EA's superior anti-biofilm activity relative to these other components (Figure 4C). This concentration-dependent efficacy, combined with EA's significantly greater abundance in CSR, led us to conclude that EA serves as the predominant antifungal component in CSR against *C. albicans*. The experimental design prioritized EA based on these quantitative and activity differences.

9. Additionally, the authors should state the exact concentration of EA used when added to CSR (i.e., the equivalent amount mentioned in the experiments).

**Response:** Thank you for the comments. We have specified the exact concentration of EA (4 μg/mL) used when added to CSR in the section titled “Validation of the pharmacological substance ellagic acid” on line 343.

10. In Figure 4D, there does not appear to be a significant visual difference in the microscopic images of the biofilms treated with CSR versus CSR+EA. To validate these findings, the authors are encouraged to repeat the experiment or include quantitative evidence to support the visual data.

**Response:** Thank you for the comments. The microscopic images in Figure 4D were intended to illustrate morphological changes in *C. albicans* biofilms following treatment with CSR and CSR+EA. As shown in Figure 4E (originally 4D), the hyphal structures of *C. albicans* were nearly eliminated by both treatments. To avoid potential misunderstanding from the data presentation in the original Figure 4D, we have rearranged the figure panels, moving the mycelial structure diagram originally presented in original 4D to Figure 4F for clearer representation.

Reviewer #3:

Authors have explored an interesting topic but data presented in this study is either missing, questionable or irreproducible. Poor scientific writing, inappropriate methodology, results, discussion and graphical representation of the data. Hence, the study is unsuitable for the publication. Authors need to rethink the motive behind this study and rewrite, re-analyze, re-perform experiments.
Few comments have been mentioned as a comment in attached PDF file.

**Response:** We thank Reviewer 3 for the thorough and constructive comments. In response, we have carefully rewritten the manuscript for clarity and improved scientific writing. Additionally, we have re-analyzed the data and re-performed key experiments to ensure the reliability and reproducibility of our findings. We believe these revisions have significantly strengthened the quality of the study.

Reviewer #4: This manuscript presents a study investigating the enhanced antifungal properties of Charred Sanguisorbae Radix (CSR) against Candida albicans, focusing on yeast-to-hypha differentiation and biofilm formation. While the research is based on an interesting traditional medicine approach, it suffers from several methodological and interpretational weaknesses that limit its scientific rigor and clinical relevance. Some concerns pertaining to the study are listed below:

1. While clotrimazole is used as a control, the manuscript does not sufficiently compare CSR’s efficacy with clinically relevant concentrations of standard antifungals (e.g., fluconazole or echinocandins. Also, clotrimazole usage as positive control is not consistent across experiments.

**Response:** We thank Reviewer 4 for the comments. The MIC of fluconazole extract against *C. albicans* was 15.6 ug/ml, as presented in S2 Table. In updated S1_Fille, we supplemented fluconazole as a positive control *in vivo*. As shown in S5 Fig, after fluconazole treatment, there was no significant keratinization or congestion in the submucosal tissue and marked improvement in inflammation. However, some hyphae remained attached to the vaginal wall.

2. Quantitative metrics (MICs, biofilm IC50 and inhibition percentages) are missing and phrases like strong inhibition were used. Quantitative representations enhance reader comprehension on the efficacy of CSR.

Response: Thank you for the comments. Phrases like strong inhibition have been clarified or removed where appropriate.

3. Although CSR inhibits hyphal transition, it fails in fungal burden elimination especially in comparison to clotrimazole which is a serious drawback. How do the authors justify clinical relevance in this case?

**Response:** Thank you for the insightful comment. We acknowledge the limitation that CSR does not achieve fungal burden elimination comparable to clotrimazole. However, it is important to note that the yeast form of *C. albicans* is generally non-pathogenic, and its transition to the hyphal form is a critical step in the development of pathogenicity [1,2]. This morphological switch facilitates the expression of key virulence factors, including those involved in adhesion, invasion, and biofilm formation as hallmarks of persistent and treatment-resistant infections. Moreover, biofilm formation represents a major clinical challenge, as *C. albicans* within biofilms exhibit significantly reduced susceptibility to antifungal agents. CSR’s strong inhibitory effect on hyphal transition and biofilm formation suggests it may play a valuable role in preventing or mitigating infection progression, particularly in cases where biofilm-associated resistance is a concern. Therefore, despite its limited impact on total fungal burden, we believe CSR holds clinical relevance as an adjunct or alternative strategy targeting virulence and persistence mechanisms of *C. albicans*.

References:

[1] Jacobsen ID, Wilson D, Wächtler B, Brunke S, Naglik JR, Hube B. Candida albicans dimorphism as a therapeutic target. Expert Rev Anti Infect Ther. 2012;10(1):85-93.

[2] Priya A, Pandian SK. Piperine Impedes Biofilm Formation and Hyphal Morphogenesis of Candida albicans. Front. Microbiol.2020;11:756.

4. The study lacks pharmacokinetic or toxicity studies of CSR or EA in vivo, which limits claims about therapeutic potential. Cytotoxicity data of CSR on mammalian cells are not provided, raising safety concerns for clinical application. Also, charring and carbonizing can produce carcinogens which can be detrimental to clinical application. The authors should address these issues.

**Response:** We appreciate these valid safety considerations regarding CSR's clinical potential. While our current study focused on *in vitro* antifungal activity using pharmacopeia-standard carbonized material (150-300°C charcoal roasting), we acknowledge that *in vivo* pharmacokinetic and toxicity studies would strengthen therapeutic claims. Chemical characterization of our CSR extract revealed no detectable carcinogens in previous work, consistent with known carbonization effects that convert complex tannins into safer phenolic derivatives [1]. The Chinese Pharmacopeia mandates external use only for CSR, and prior research demonstrates processed Sanguisorba exhibits reduced cytotoxicity versus crude form [1]. However, we agree future work should include mammalian cell cytotoxicity assays, in vivo safety profiling, and thorough characterization of carbonization byproducts. These steps will better establish CSR's therapeutic window while maintaining its traditional safety profile as an approved topical agent.

Reference:

[1] Wang Z, Yang C, Wu L, Sun J, Wang Z, Wang Z. Variation of Saponins in Sanguisorba officinalis L. before and after Processing (Paozhi) and Its Effects on Colon Cancer Cells In Vitro. Molecules. 2022;27(24):9046.

**Chemical Composition Identification Results of CSR**

| No. | RT | m/z reality | m/z theory | ppm | Molecular formula | Compounds | CAS | Ionization mode | Compound category |
| --- | --- | --- | --- | --- | --- | --- | --- | --- | --- |
| 1 | 0.75 | 331.0671 | 331.06707 | 1.9 | C_13_H_16_O_10_ | 1-O-Galloyl-β-D-glucose,β-glucogallin | 13405-60-2 | -H | Hydrolyzable tannins |
| 2 | 1.16 | 169.0142 | 169.01425 | 2.1 | C_7_H_6_O_5_ | gallic acid | 149-91-7 | -H | Hydrolyzable tannins |
| 3 | 1.49 | 125.0244 | 125.02442 | -4.1 | C_6_H_6_O_3_ | pyrogallol | 87-66-1 | -H | Hydrolyzable tannins |
| 4 | 2.21 | 153.0193 | 153.01933 | -0.9 | C_7_H_6_O_4_ | protocatechuic acid | 99-50-3 | -H | Hydrolyzable tannins |
| 5 | 2.48 | 345.0827 | 345.08272 | 2.3 | C_14_H_18_O_10_ | methyl 6-O-galloyl-β-D-glucopyranoside | / | -H | Hydrolyzable tannins |
| 6 | 2.69 | 183.0299 | 183.0299 | 1.1 | C_8_H_8_O_5_ | methyl gallate | 99-24-1 | -H | Hydrolyzable tannins |
| 7 | 3.16 | 137.0244 | 137.02442 | 1.3 | C_7_H_6_O_3_ | 3,4-dihydroxy-benzaldehyde | 139-85-5 | -H | Hydrolyzable tannins |
| 8 | 3.72 | 291.0863 | 291.08631 | 3 | C_15_H_14_O_6_ | (+)-catechin | 154-23-4 | +H | Flavonoids |
| 9 | 4.73 | 469.0049 | 469.00486 | 4.8 | C_21_H_10_O_13_ | sanguisorbic acid dilactone | 82203-11-0 | -H | Hydrolyzable tannins |
| 10 | 5.91 | 197.0455 | 197.04555 | 0.8 | C_9_H_10_O_5_ | methyl 3-O-methyl-gallate | / | -H | Hydrolyzable tannins |
| 11 | 5.91 | 197.0455 | 197.04555 | 0.8 | C_9_H_10_O_5_ | ethyl gallate | 831-61-8 | -H | Hydrolyzable tannins |
| 12 | 6.57 | 300.999 | 300.99899 | 2.4 | C_14_H_6_O_8_ | ellagic acid | 476-66-4 | -H | Hydrolyzable tannins |
| 13 | 9.97 | 213.0758 | 213.07575 | 5.4 | C_10_H_12_O_5_ | 3, 4-dihydroxy-5-methoxybenzoic acid ethyl ester | 78533-49-0 | +H | Hydrolyzable tannins |
| 14 | 9.97 | 213.0758 | 213.07576 | 5.4 | C_10_H_12_O_5_ | 3,4-dimethoxy-5-hydroxybenzoic acid methyl ester | 83011-43-2 | +H | Hydrolyzable tannins |
| 15 | 10.73 | 329.0303 | 329.03029 | 4.9 | C_16_H_10_O_8_ | 3,4′-O-dimethylellagic acid | / | -H | Hydrolyzable tannins |
| 16 | 10.77 | 303.0499 | 303.04993 | 3.2 | C_15_H_10_O_7_ | quercetin | 117-39-5 | +H | Flavonoids |
| 17 | 13.37 | 255.0652 | 255.06519 | 3.2 | C_15_H_10_O_4_ | 1,8-dihydroxy-3-methylanthraquinone | 481-74-3 | +H | Other |
| 18 | 18.33 | 485.3272 | 485.32725 | 3.8 | C_30_H_46_O_5_ | rosamultic acid | 214285-76-4 | -H | Triterpenoids |
| 19 | 19.46 | 483.3116 | 483.3116 | 5.2 | C_30_H_44_O_5_ | fupenzic acid | 119725-20-1 | -H | Triterpenoids |
| 20 | 19.77 | 487.3429 | 487.3429 | 5.5 | C_30_H_48_O_5_ | tormentic acid | 13850-16-3 | -H | Triterpenoids |
| 21 | 19.77 | 487.3429 | 487.3429 | 5.5 | C_30_H_48_O_5_ | arjunic acid | 31298-06-3 | -H | Triterpenoids |
| 22 | 21.98 | 469.3323 | 469.33233 | 4.2 | C_30_H_46_O_4_ | 3-oxo-19α-hydroxyurs-12-en-28-oic acid | 13849-90-6 | -H | Triterpenoids |
| 23 | 23.82 | 457.3676 | 457.36762 | 4.5 | C_30_H_48_O_3_ | Ursolic acid | 77-52-1 | +H | Triterpenoids |
| 24 | 25.4 | 469.3323 | 469.33233 | 3.3 | C_30_H_46_O_4_ | 2α,3β-dihydroxyurs-12,18-dien-28-oic acid-28-O-β-D-glucopyranosyl ester | / | -H | Triterpenoids |
| 25 | 26.72 | 453.3374 | 453.33742 | 5 | C_30_H_46_O_3_ | 3β-hydroxyurs-12, 19(20)-dien-28-β-D-glucopyranoside | 6812-98-2 | -H | Triterpenoids |

5. The justification for using CSR based on traditional Chinese medicine is not well-supported by modern pharmacological rationale. Some references are outdated or rely heavily on non-peer-reviewed Chinese sources. Also, some claims and statements pertaining to TCM are missing in the introduction.

Response: Thank you for the comments. We have added more recent publications using CSR to further support the rational of study. The new reference [22] is added in the introduction section on lines 93-95.

6. While gene expression changes are reported, causal links between these gene alterations and CSR’s effects are assumed but not experimentally validated. The role of CSR and EA vs PYG in modulating specific pathways is not dissected. A more holistic discussion on the interplay of gene expression changes and how CSR modulates these metabolic pathways to possibly interpret a mode of action should be done.

**Response:** Thank you for the valuable comments. In response, we have expanded the Discussion section (lines 485-506) to include a more holistic analysis of the gene expression changes observed. We now provide a broader interpretation of how CSR may modulate key metabolic and virulence-related pathways. While we acknowledge that the causal links between specific gene alterations and the effects of CSR and EA (versus PYG) have not been directly validated in this study, the revised discussion explores the potential mechanisms of action based on transcriptomic trends and relevant literature. These insights offer a foundation for future studies aimed at dissecting the molecular pathways involved.

7. Error bars and exact p-values are not clearly reported. Some graphs show error bars values comparable to the actual value (Fig. 1B) but authors report it as significant. The authors are advised to redo the statistical analysis and check the graph representations.

**Response:** Thank you for the comments. We have re-conducted the statistical analyses and carefully re-examined the graphical representations, including Fig 1B. Error bars have been corrected to accurately reflect the data variability, and exact p-values have been reported where appropriate. The updated Fig 1 and statistical results have been revised accordingly to ensure clarity and accuracy.

8. The study focuses solely on VVC and broader implications for other Candida infections are speculative as Candida manifests in other routes like oral, skin etc.

**Response:** Thank you for the comments. Previous studies have shown that ellagic acid inhibited *Candida parapsilosis* and *C. neoformans* more effectively than *Candida krusei* and also some *Candida albicans* clinical strains [1]. In addition, Ellagic Acid-Cyclodextrin Complexes has antifungal and anti-inflammatory effects, reducing the invasive capacity of *C. albicans*, which suggests that EA/HP-β-CD may be a promising alternative for the treatment of oral candidiasis [2]. Therefore, broader implications of CSR for other Candida infections are speculative.

References:

[1] Rangkadilok N, Tongchusak S, Boonhok R, Chaiyaroj SC, Junyaprasert VB, Buajeeb W, et al. In vitro antifungal activities of longan (Dimocarpus longan Lour.) seed extract. Fitoterapia. 2012;83(3):545-53.

[2] Sampaio ADG, Gontijo AVL, Lima GMG, de Oliveira MAC, Lepesqueur LSS, Koga-Ito CY. Ellagic Acid-Cyclodextrin Complexes for the Treatment of Oral Candidiasis. Molecules. 2021;26(2):505.

9. The study lacks vehicle control (e.g., ethanol or DMSO without extract) to ensure that observed effects are due to CSR and not solvents.

**Response:** In all experiments, the control groups were treated with DMSO alone (without extract) to account for any potential solvent effects. This information has now been added to the ‘Methods’ section on lines 205, 225, 238, 245, 342.

10. The specificity of CSR and EA effects on Candida albicans vs. host cells or other microbial species is not addressed

**Response:** Thank you for the comments. CSR and EA exhibit antimicrobial activity. There have been reports indicating that CSR exhibits antimicrobial activity against *Staphylococcus aureus* [1] and *Trichophyton rubrum* [2]. Additionally, EA demonstrates efficacy against *Helicobacter pylori* [3], *Trichophyton rubrum* [4], and *Candida auris* [5]. We have added this information to the Discussion section on lines 559-563.

References:

[1] Gu W, Yao JH, Lu MM, Su M, Zou JQ, Cai XY, et al. Effect of processing technology on antibacterial effect of Sanguisorbae Radix based on theory of “maintaining medicinal properties aftercarbonisatus”. Chinese Herb. Med. 2022;53(04):1042-50.

[2] Huangjin Tong, Man Su, Xuxi Cheng, Hongdan Wu, Qinglian Hu, Huiwen Yu, et al. Enhanced antibacterial effect and mechanism of Sanguisorbae Radix after carbonization against Tricho-phyton rubrum. Chinese Journal of Hospital Pharmacy, 2024, 44(24): 2845-2851.

[3] De R, Sarkar A, Ghosh P, Ganguly M, Karmakar BC, Saha DR, et al. Antimicrobial activity of ellagic acid against Helicobacter pylori isolates from India and during infections in mice. J Antimicrob Chemother. 2018;73(6):1595-1603.

[4] Li ZJ, Guo X, Dawuti G, Aibai S. Antifungal Activity of Ellagic Acid In Vitro and In Vivo. Phytother Res. 2015;29(7):1019-1025.

[5]	Possamai Rossatto FC, Tharmalingam N, Escobar IE, d'Azevedo PA, Zimmer KR, Mylonakis E. Antifungal Activity of the Phenolic Compounds Ellagic Acid (EA) and Caffeic Acid Phenethyl Ester (CAPE) against Drug-Resistant Candida auris. J Fungi (Basel). 2021;7(9):763.

11. The study claims EA is the primary active compound but does not test EA alone in the mouse model to confirm this in vivo. Also, discussion on pros and cons of using CSR vs EA alone is missing in the discussion.

**Response:** Thank you for the insightful comment. We acknowledge the limitation that EA was not tested independently in the mouse model, which prevents us from fully confirming it’s *in vivo* efficacy as the primary active compound. This is an important experiment that we plan to conduct in future studies. In response to your comment, we have also added a discussion on the potential advantages and limitations of using CSR extract versus EA alone in the revised Discussion section (lines 550-553), highlighting considerations such as compound synergy, bioavailability, and translational potential.

12. The study focuses solely on one Candida albicans strain. There’s no testing on clinical isolates, other Candida species (C. glabrata, C. krusei), or biofilm-competent strains with higher resistance profiles. Broad-spectrum antifungal activity is claimed based on past SR studies, but not directly demonstrated here.

**Response:** Thank you for the comment. We acknowledge that the current study is limited to a single *C. albicans* strain, and we did not include clinical isolates or other Candida species such as *C. glabrata* or *C. krusei*. At present, we do not have access to resistant clinical isolates; however, we are actively working to collect these strains for future studies. This will allow us to further validate the antifungal spectrum and efficacy of CSR, particularly against biofilm-competent and drug-resistant strains.

13. Herbal medicines are subject to batch-to-batch variability in active compounds. There is no discussion or data on the standardization of CSR extracts.

**Response:** We appreciate this important observation regarding herbal medicine standardization. Our study evaluated different batches of CSR showing ellagic acid (EA) content variability (3.3%-7.1%), which we've acknowledged as a study limitation (Lines 564-568). For quality control, we followed Chinese Pharmacopoeia standards using gallic acid (GA) content (≥0.60% for CSR vs ≥1.0% for SR), while our samples complied as CSR:2.9%; SR:1.8%. Additionally, our prior analysis of 12 batches revealed ranges for: GA (crude:0.5-1.3%; carbonized:0.8-1.7%), EA (crude:0.3-0.7%; carbonized:3.3-7.1%), and PYG (crude:0.02-0.06%; carbonized:0.2-0.9%) (PMID: 35309515). Notably, extract concentrations exceed crude slice levels, making direct pharmacopoeia comparisons invalid. These findings underscore both the need for standardized extraction protocols and the importance of establishing extract-specific quality standards.

The references are as follows:

1. Wei Gu, Hao Wang, Man Su, Yiwei Wang Fei Xu, Qinglian Hu, Xuyi Cai, Jinyun Song, Huangjin Tong, Yuerong Qian, Hongyu Zhao, Jun Chen*. Investigation of tannins transformation in Sanguisorbae Radix over carbonizing by stir-frying[J]. Front Mol Biosci. 2022 Mar 2;9:762224.
2. Cheng XX, Zhou ZY, Hu QL,Wu HD, Qi SY, Li XX, et al. Charcoal-frying process and quality markers of Sanguisorbae Radix based on hemostatic effect. China J. Chin. Mater. Med. 2024;49(10):2710-2721.
3. Gu W, Yao JH, Lu MM, Su M, Zou JQ, Cai XY, et al. Effect of processing technology on antibacterial effect of Sanguisorbae Radix based on theory of “maintaining medicinal properties aftercarbonisatus”. Chinese Herb. Med. 2022;53(04):1042-1050.
14. Biofilm quantification relies heavily on crystal violet staining, which measures total biomass but not viable cell counts or metabolic activity (e.g., via XTT or resazurin assays). Also, confocal microscopy and comstat should be incorporated to better evaluate biofilm architectures and provide a more accurate quantification in comparison to crystal violet.

**Response:** Thank you for the insightful comment. In addition to crystal violet staining, we have performed confocal laser scanning microscopy and quantitative analysis using COMSTAT to better assess biofilm architecture. These results are now presented in Fig 2B and 2C, providing a more accurate and detailed evaluation of the biofilm structure and supporting the conclusions drawn from the biomass data.

15. No functional validation (e.g., knockout strains) was done to confirm that changes in gene expression are causative for biofilm inhibition.

**Response:** Thank you for the comment. We agree that functional validation, such as using knockout or overexpression strains, would be essential to confirm whether the observed gene expression changes are causally linked to biofilm inhibition. While this was beyond the scope of the current study, we recognize it as a critical direction for future research and have noted this limitation in the revised Discussion section on lines 568-572.

16. Grammatical errors and awkward phrasing detract from the manuscript's clarity. Phrases like "CSR caused the least pathological damage" for example should be avoided. Some scientific terms are misused or loosely applied (e.g., “biofilm dispersal phase” is not clearly defined). The authors should review and revise the manuscript in its entirety to adhere to academic writing standards. Alternatively, they should employ language editing services to enhance manuscript interpretation.

**Response:** Thank you for the comments. We appreciate the reviewer’s feedback regarding the language and terminology used in the manuscript. In response, we have carefully revised the entire manuscript to improve clarity, correct grammatical errors, and ensure the accurate use of scientific terminology. Phrases such as “CSR caused the least pathological damage” have been corrected for precision in line 377, and terms like “biofilm dispersal phase” have been clarified or removed where appropriate in lines 406-412.
